# Optically transparent multi-suction electrode arrays

**DOI:** 10.3389/fnins.2015.00384

**Published:** 2015-10-20

**Authors:** John M. Nagarah, Annette Stowasser, Rell L. Parker, Hiroki Asari, Daniel A. Wagenaar

**Affiliations:** ^1^Division of Biology, California Institute of TechnologyPasadena, CA, USA; ^2^Biological Sciences, University of CincinnatiCincinnati, OH, USA

**Keywords:** electrophysiology, multielectrode array, voltage-sensitive dye, neural interface, brain slice, retina, leech

## Abstract

Multielectrode arrays (MEAs) allow for acquisition of multisite electrophysiological activity with submillisecond temporal resolution from neural preparations. The signal to noise ratio from such arrays has recently been improved by substrate perforations that allow negative pressure to be applied to the tissue; however, such arrays are not optically transparent, limiting their potential to be combined with optical-based technologies. We present here multi-suction electrode arrays (MSEAs) in quartz that yield a substantial increase in the detected number of units and in signal to noise ratio from mouse cortico-hippocampal slices and mouse retina explants. This enables the visualization of stronger cross correlations between the firing rates of the various sources. Additionally, the MSEA's transparency allows us to record voltage sensitive dye activity from a leech ganglion with single neuron resolution using widefield microscopy simultaneously with the electrode array recordings. The combination of enhanced electrical signals and compatibility with optical-based technologies should make the MSEA a valuable tool for investigating neuronal circuits.

## 1. Introduction

Multielectrode arrays (MEAs, Gross et al., 1977; Gross, 1979; Pine, 1980; Obien et al., 2014) have successfully been used to study a range of preparations including dissociated cortical cultures (Wagenaar et al., 2006), retinal explants (Meister et al., 1991), and hippocampal slices (Steidl et al., 2006). Obtaining strong voltage signals from most of these preparations is possible, but slices continue to pose challenges, likely because of the presence of a layer of dead cells at the surface of the slice and limited oxygenation through the slice to the living cells nearest to the array. The introduction of perforated MEAs in polyimide is an improvement in regard to tissue oxygenation and signal to noise ratio, but has limitations: Whereas glass-based MEAs are transparent (except for the electrodes and sometimes the leads) (Gross et al., 1985), presently available commercial perforated MEAs are not due to the translucent polyimide substrate (Egert et al., 2005). In an age in which combination of MEAs with optical methods such as voltage- or calcium-sensitive dyes (Peterka et al., 2011; Gaudry and Kristan, 2012) or optogenetics (Tye and Deisseroth, 2012) becomes ever more desirable, this is a major disadvantage.

**Figure 1:**
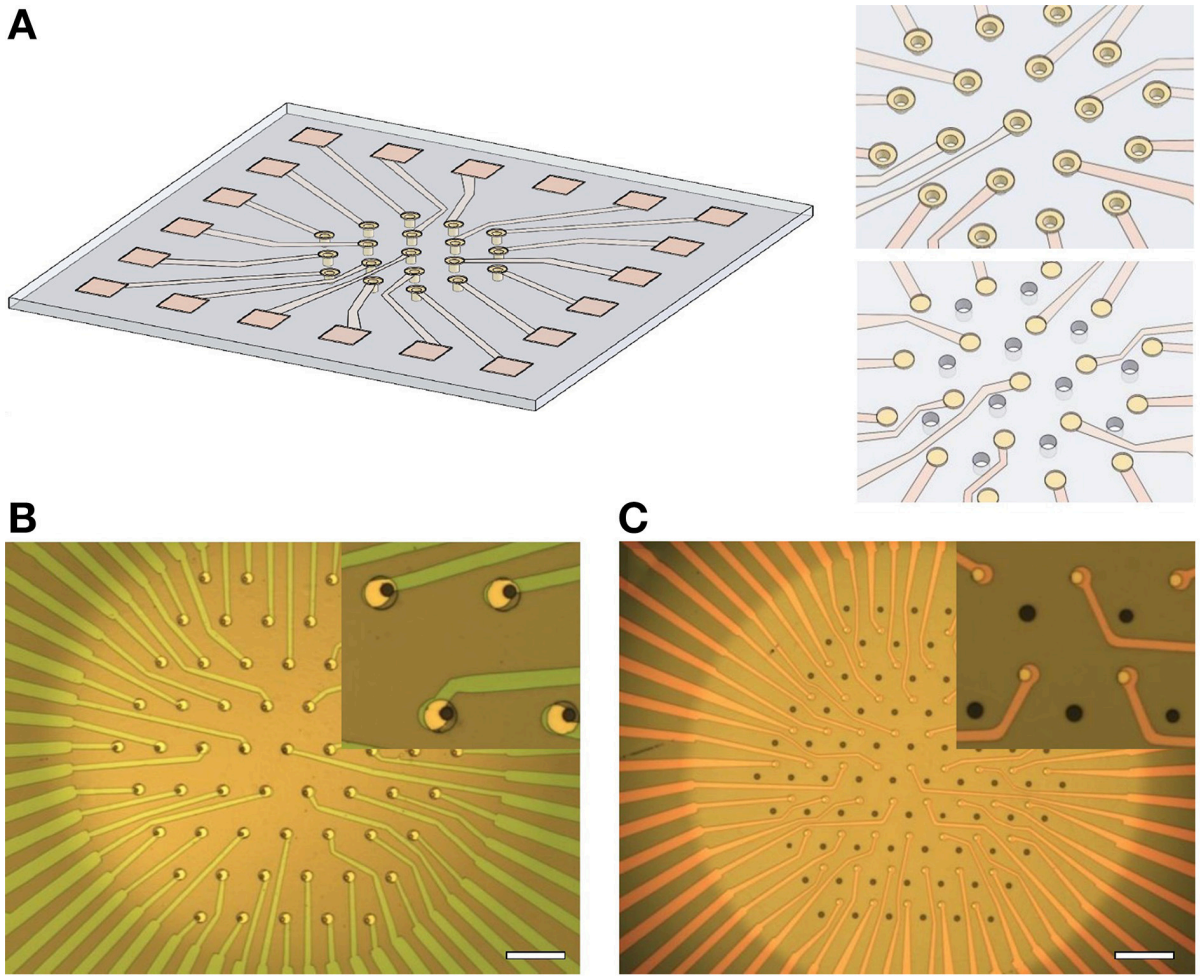
**Overview of multisuction electrode array (MSEA) technology**. **(A)** Artist's impression of a multi-electrode array with suction pores embedded within each electrode. Insets: Close-up of electrodes with embedded pores (top) and alternative layout with pores between electrodes (below). **(B)** Visible light image of MSEA with detail at larger scale (inset). Scale bar: 100 μm. **(C)** Visible light image of MSEA with suction pores between electrodes. Scale as in **(B)**.

Previously, polyimide was preferred as the substrate for perforated MEAs because of the relative ease of creating micrometer-sized perforations in an organic substrate vs. a glass-based substrate (Egert et al., 2005). However, recent material processing advances in our lab have allowed for the fabrication of thinned, optically transparent suspended membranes and though-hole arrays in fused silica (amorphous quartz, Nagarah et al., 2010; Nagarah and Wagenaar, 2012). The devices we present here, multi-suction electrode arrays (MSEAs), feature electrodes placed immediately around or in between suction holes in quartz (Figure 1).

We demonstrate the practical utility of these MSEAs firstly by recording from mouse cortico-hippocampal slices, in which suction resulted in a dramatic increase in signal-to-noise ratio of recorded spikes and a concomitant increase in the number of detectable units. We then show that applied suction also substantially improves the quality of recordings from mouse retina, a classic example of a preparation for which MEA recordings have yielded important scientific results (Meister et al., 1991; Ryan et al., 2013). Lastly, in a demonstration experiment of their compatibility with optical recording techniques, we use an MSEA to record from the nervous system of the medicinal leech, in which a voltage-sensitive dye provides simultaneous confirmation of intracellular electrical activity.

## 2. Materials and methods

### 2.1. MSEA fabrication

**Figure 2:**
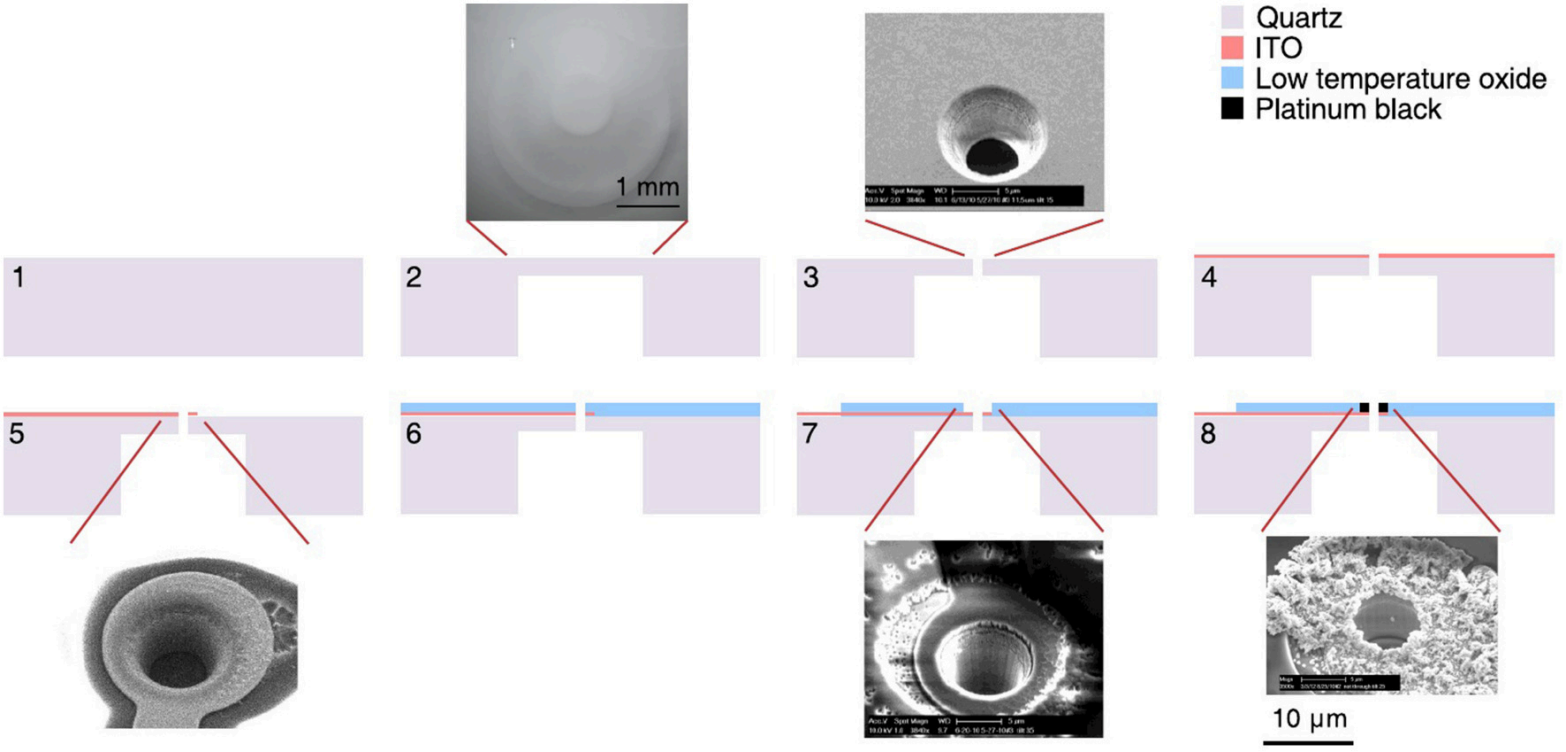
**MSEA fabrication process flow**. 1, Clean fused silica slide; 2, deep wet etching into fused silica; 3, etching 10–15 μm diameter suction pores into suspended fused silica membrane; 4, sputtering thin indium tin oxide (ITO) film on top of device; 5, patterning and etching leads and electrodes into ITO; 6, deposition of silicon dioxide film on top of device to electrically insulate ITO; 7, patterning and etching silicon dioxide to expose ITO contacts and electrodes; 8, electroplating platinum black onto ITO electrodes. Scale bar in step 8 applies to all four SEM images.

The fabrication flow is summarized in Figure 2. Fused silica slides, 50 × 50 mm with a thickness of 0.2 or 0.5 mm were used as a substrate for the MSEA device (step 1). A trench was etched in the backside of the substrate resulting in a 10–15 μm thick suspended membrane (step 2, Nagarah and Wagenaar, 2012). This allowed through-pores, 10–15 μm in diameter, to be plasma etched into the fused silica membrane (step 3, Nagarah et al., 2010). A transparent 100-nm-thick indium tin oxide (ITO) film was sputtered onto the front-side of the substrate with a Denton Discovery 550 sputterer (Denton Vacuum, LLC; Moorestown, NJ; step 4). The MSEA contacts, leads, and electrodes were patterned with positive photoresist and transferred into the ITO with a SLR 770 Unaxis ICP RIE (Oerlikon; Switzerland) with Cl_2_/BCl_3_ chemistry (step 5). After stripping the photoresist in PRX-127 (Rohm Haas; Philadelphia, Pennsylvania) at 70°C, a 1–1.5 μm thick low temperature oxide (LTO) film (SiO_2_ glass) was deposited in a LPCVD tube furnace (Tystar; Torrance, CA; step 6). Positive photoresist was then spin-coated onto the substrate to open the leads and electrodes through the LTO film. Before spin-coating, adhesive tape was adhered to the underside of the trench to prevent photoresist being drawn through the pores by the spin-coater vacuum chuck. The exposed LTO film was etched in an Advanced Oxide Etcher (SPTS; Newport, UK) to expose the underlying ITO (step 7). The photoresist was then stripped in PRX-127. Two designs of the MSEA were used: A “circumferential design,” in which the through pores were placed in the center of ring-shaped electrodes, and an “intercalated design,” in which the through pores were placed between disk-shaped electrodes. The electrodes were arranged in a hexagonal lattice. The circumferential electrodes were 10–15 μm in diameter with 87.5 μm spacing between electrodes within a row and between rows (Figure 1B). The intercalated electrodes were 10 μm in diameter with 70 μm spacing (Figure 1C).

To lower the ITO electrochemical impedance in saline solution, Pt black was electroplated onto the exposed ITO electrodes (step 8): A Pt chloride solution (1% chloroplatinic acid hexahydrate, 0.0025% hydrochloric acid, 0.01% lead ascorbate in water) was used as an electroplating solution (Maher et al., 1999). Before plating, the MSEA was exposed to an oxygen plasma to clean the exposed electrodes. Current was sourced through the ITO electrode with a 2.2-MΩ resistor parallel to ground and a Pt wire as a counter electrode. Platinum black was electroplated by pulsing current: –0.4 μA for 2 s, 0 μA for 8.5 s, 10 cycles. To minimize breakage of Pt spines during an experiment, the electrodes were immersed in 30% hydrogen peroxide so the oxygen bubbling action from the Pt electrode will preventively break the weak platinum spines beforehand (Maher et al., 1999). This plating-peroxide treatment was conducted a total of five times.

### 2.2. Tissue preparation

All experimental procedures on mice were carried out in accordance with protocols approved by the Institutional Animal Care and Use Committee at the California Institute of Technology.

### 2.3. Mouse cortico-hippocampal slice

Mice used for recordings were wild-type, aged P8–P17, sex undetermined. To prepare slices, animals were anesthetized with CO_2_ and then decapitated. The brain was dissected out, placed in ice-cold ACSF for a few minutes for initial cooling and then mounted on a vibratome. Coronal sections (250 μm) were made using a Vibratome (DTK-1000; Ted Pella, Redding, CA). Slices were allowed to recover for 1 h in ACSF bubbled with 95% O_2_/5% CO_2_ at 32°C, then moved to room temperature. The ACSF consists of, in mM: 124 NaCl, 3 KCl, 1.25 NaH_2_PO_4_, 26 NaHCO_3_, 10 glucose, 1.3 MgSO_4_, and 2.5 CaCl_2_. After 15 min at room temperature, the slices were put into fresh room temperature ACSF. Recordings were made in a chamber perfused with ACSF at 32°C, bubbled with 95% O_2_/5% CO_2_, at a rate of 1–2 mL/min.

### 2.4. Mouse retina explant

Mice retinae were prepared as previously described (Lefebvre et al., 2008). Briefly, mice were euthanized and retinae dissected out under infrared illumination to preserve dark adaptation. As much vitreous humor was removed as practical. A piece of retina was placed with ganglion cells facing down on an MSEA on the stage of an upright microscope. The preparation was continuously superfused with Ames' medium (A1420; Sigma Aldrich, St Louis, MO) equilibrated with 95% O_2_ and 5% CO_2_ gas at room temperature.

### 2.5. Leech nervous system

Leeches (*Hirudo verbana*) were maintained as previously reported (Harley et al., 2011). Nerve cords were extracted following the procedures of Baljon and Wagenaar (2015), except that here either a single ganglion or the entire nerve cord (sans the head brain, which would inhibit fictive swimming) was extracted rather than a short chain of ganglia. The leech nervous system contains embedded muscle cells that tend to cause strong motion artifacts. To prevent these and to ensure contact with the entire curved surface of a ganglion, ganglia were immobilized on a slab of polydimethylsiloxane (PDMS; Sylgard 184; Dow Corning Corporation; Midland, MI). Ganglia were always desheathed before recording.

For leech tissue electron microscopy, desheathed ganglia were fixed with osmium tetroxide and dehydrated using a graded series of ethanol. The ganglia were then immersed in t-butyl alcohol and frozen (4°C). The t-butyl alcohol was sublimed under vacuum to dry the ganglia (Inoue and Osatake, 1988).

### 2.6. MSEA recordings

A custom-molded PDMS solution reservoir was adhered onto the MSEA before filling with saline solution. A custom-built acrylic o-ring holder was used to apply suction from the underside of the MSEA substrate.

Cortico-hippocampal slices were transferred into the recording chamber with a wide-mouth pipette and positioned over the array with a small paint brush. Slices were immobilized over the array using a harp-style anchor (64-1418, Warner Instruments; Hamden, CT), with care taken to keep harp strings away from the recording area. In select experiments, slices were electrically stimulated using monophasic voltage pulses of up to 600 mV amplitude and 100 μs duration applied between adjacent electrodes.

Mouse retinae were transferred into the recording chamber in the same way. Since the mouse retina explant has strong natural curvature, a custom-built harp with a stainless steel rim and wide nylon mesh was used to keep the retina flat over the array with minimal force. Retinae were stimulated with full-field amber illumination using a LED (LED-30W0310, SatisLED; Shenzhen, China) combined with an interference filter (HQ590/60m, Chroma Technology Corp.; Bellows Falls, VT) at a rate of 0.5 Hz and 50% duty cycle.

Leech ganglia were placed over the array as follows: The ganglion or nerve cord was immobilized on a PDMS slab. This slab was then attached to a micromanipulator and lifted up out of its saline solution while pulling along a large drop of saline to keep the tissue covered in liquid. While held in the manipulator, the slab was translated to the recording area, and coarsely positioned in the bath solution over the electrode array. Finally, the ganglion was gradually lowered until it touched the electrodes. One dorsal posterior nerve root (Kristan et al., 1974) was aspirated into a glass suction electrode. Brief trains of stimuli (0.5–1.0 V, 50 ms, 10 Hz) were used to evoke fictive swimming.

A MultiChannel Systems 60 channel amplifier (Model 1060) was used to record from each MSEA electrode. MC Rack software was used for the hippocampus slice recordings. For the mouse retina and leech nerve cord recordings, voltage signals were recorded with MEABench (Wagenaar et al., 2005).

### 2.7. Voltage-sensitive dye recordings

The voltage-sensitive dye VF2.1.Cl was prepared according to standard protocol (Miller et al., 2012) and applied to the target ganglion by perfusing for 20 min using a peristaltic pump (Cacciatore et al., 1999). A custom dual upright and inverted microscope was used for imaging. It comprised an Olympus upright BX WI fluorescence train mounted atop an Olympus inverted IX51 microscope (Olympus America, Inc., Center Valley, PA) with a custom-built stage. The upright microscope was used for specimen placement over the array while the inverted objective (Olympus 20x, NA 1.0) was used for fluorescent imaging through the transparent MSEA. Illumination was provided at 460 nm with a 5-W LED light (LedEngin, San Jose, CA) that was stabilized using optical feedback (Wagenaar, 2012). We used a 460/50 nm band-pass excitation filter, 505 nm dichroic mirror, and 510 nm long pass emission filter (Chroma Technology Corporation). Images were recorded with a cooled CCD camera (QuantEM 512SC; Photometrics, Phoenix, AZ) and custom software.

### 2.8. Data analysis

All fluorescent imaging data and electrode MSEA recordings were analyzed using custom Octave (Eaton et al., 2014) scripts. The threshold for spike detection was set at 5x estimated RMS noise. Spike sorting was accomplished with UltraMegSort2000 (Fee et al., 1996; Hill et al., 2011) with standard parameters. The cross-correlations of the firing rates of the sources in **Figures 6B,C** represent the maximum peak in the cross-correlogram with 1 ms bins extended to a maximum time lag of 50 ms. The coherence plot in **Figure 8B** was calculated using a previously described method (Taylor et al., 2003). All graphs were generated using QPlot (Wagenaar, 2014).

### 2.9. Electrochemical impedance spectroscopy

Electrode impedance measurements were recorded from an MSEA with an intercalated electrode design with a VMP2 potentiostat and analyzed with EC-Lab software (BioLogic; Claix, France). We applied a sinusoidal voltage with amplitude 10 mV centered at the open circuit voltage while recording impedance between 0.1 Hz and 10 kHz. Chlorinated silver wires were used for counter and reference electrodes. The values for the circuit elements in the theoretical model in **Figure 7A** are *R*_sol_ = 100 kΩ, *R*_ct_ = 1 GΩ, *Q*_int_ = 150 nF s^*a*−1^ where *a* = 0.85, leading to a complex impedance $Z_{int}=1∕\left(i\omega^{a}Q_{int}\right)$. In **Figure 7C**, each recorded point represents the average of 10 recordings at a given frequency.

## 3. Results

### 3.1. Demonstration of basic functionality

We fabricated two types of MSEAs, one with a circumferential electrode design and one with an intercalated electrode design (Figure 1). We tested our MSEAs with three distinct preparations: cortico-hippocampal slices from mice, explanted mouse retinae, and isolated leech ganglia to determine, first of all, whether neuronal activity could be observed successfully, and secondly, whether applying suction made a difference to the quality of the recordings.

### 3.2. Cortico-hippocampal slices

**Figure 3:**
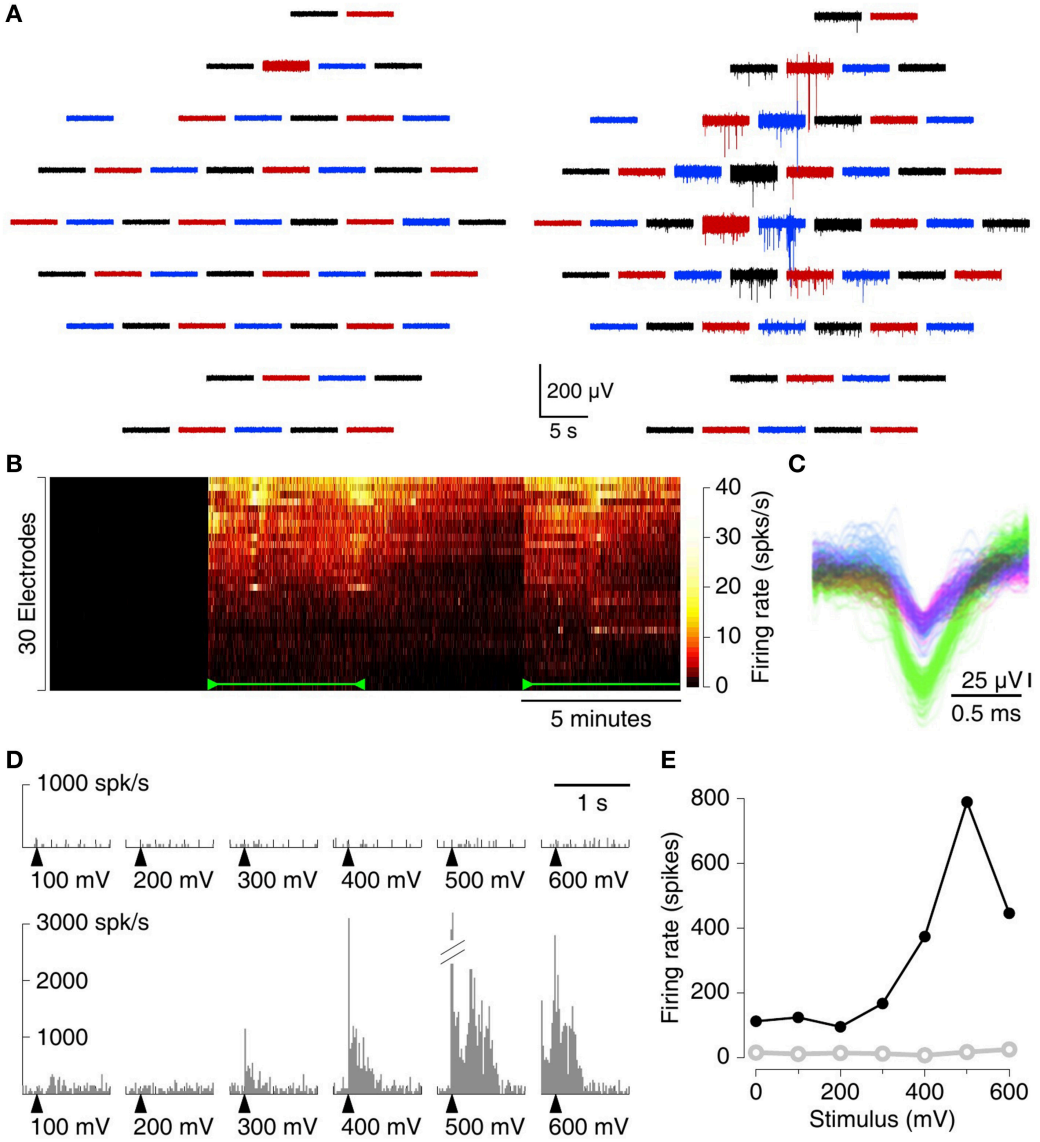
**Recording spontaneous activity from cortico-hippocampal slices with an MSEA with an intercalated electrode design**. **(A)** Simultaneously recorded traces from 60 electrodes before (left) and after (right) application of 80 mbar of suction. Each trace represents data from one electrode; traces in the graph are laid out in the same geometry as electrodes in the array. Colors only serve to guide the eye. **(B)** Heat map of firing rates on each of 30 electrodes (sorted in order of total spike count) with and without suction. Suction (110 mbar) was applied 5 min after the start of the recording, released at 10 min, and re-applied at 15 min (green marks). In this recording, no spikes were detected prior to suction at all. **(C)** Example from a single electrode of the waveforms of all spikes. Colors: units as identified by UltraMegaSort2000 (see Section 2.8). **(D)** Peristimulus time histogram of array-wide firing evoked by stimuli of varying voltage (arrowheads), before (top), and after (bottom) application of 80 mbar of suction. **(E)** Aggregate firing rates in the first 1s following stimulation before (open, gray) and after (solid, black) application of suction.

We recorded spontaneous action potentials from the CA3 region in cortico-hippocampal slices with the MSEA with the circumferential electrode designs (see Figure S1 for a photograph). Substantial spiking activity was seen on many electrodes after suction was applied and on few if any before (Figure 3A). A heat map of firing rate vs. time further demonstrates the dramatic impact of applying suction (Figure 3B). After release of suction, detectable activity gradually declined, but only fractionally and not to pre-suction baseline levels. On many electrodes, spikes from different sources (putatively different neurons) could readily be discerned (Figure 3C). Similar results were also observed from slices recorded with MSEAs with the intercalated electrode design (Figure S2).

MEAs can be used not only for recording, but also for stimulation. We tested whether electrical stimulation with standard parameters can be used to elicit activity in slices on MSEAs, and found that indeed it can. With applied suction, stimuli evoked robust activity in a stimulation voltage–dependent manner (Figures 3D,E). Before suction, no activity was observed following stimulation, but our current setup does not allow us to conclude whether this was because no activity was evoked, or whether activity was evoked but remained undetectable.

### 3.3. Explanted retinae

We used the MSEA to record from explanted retinae from wild-type mice. Previous MEA experiments on retinae found the optimal electrode diameter to be 10 μm (Meister et al., 1991). Accordingly, we used the intercalated electrode design, because we found that suction pores with diameters of at least 10 μm were necessary to apply adequate suction to the preparation.

**Figure 4:**
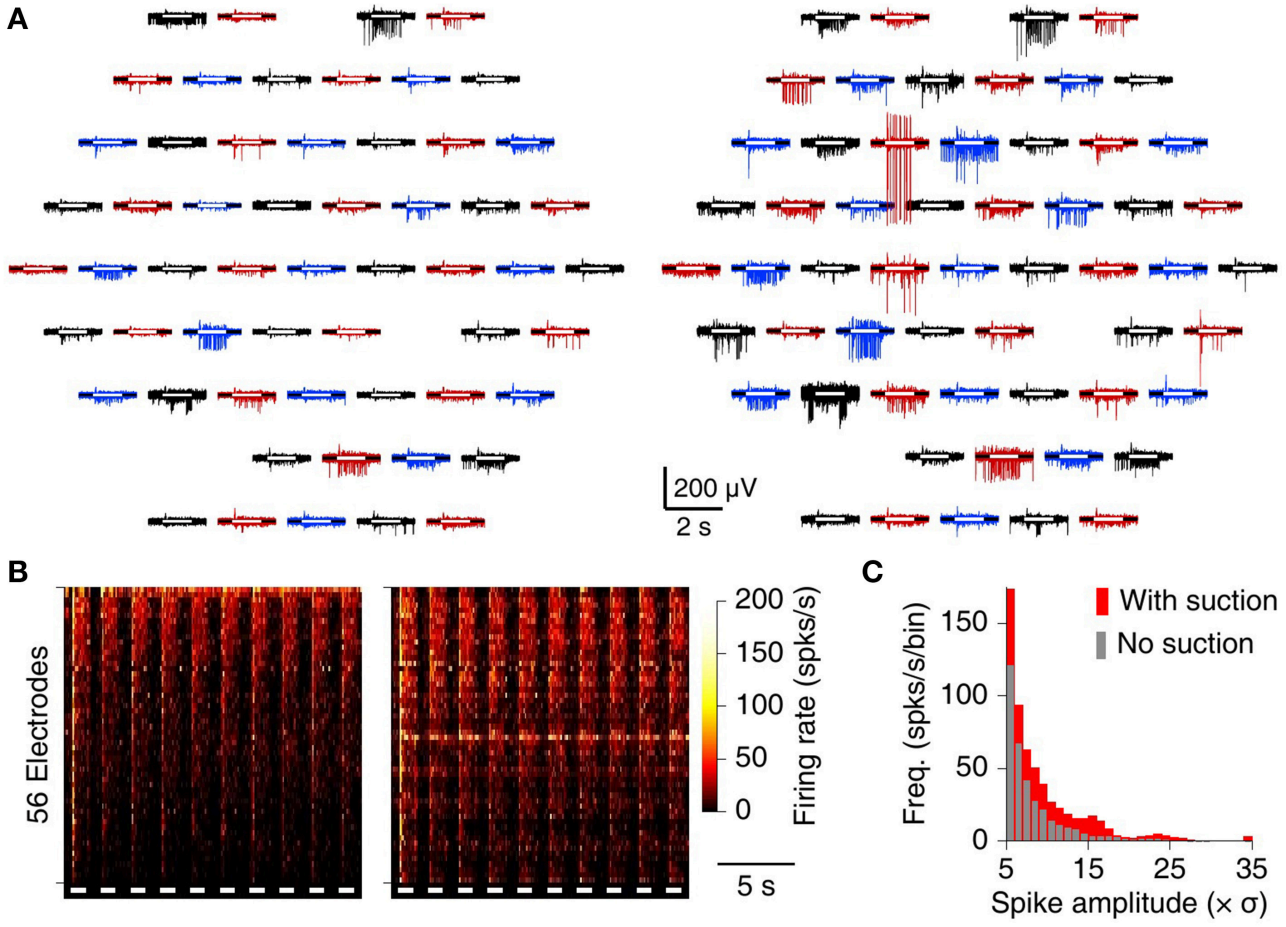
**MSEA recordings from retinal explant**. **(A)** Raw data from 60 electrodes before (left) and after (right) application of suction. White bars inside each trace indicate timing of whole-field visual stimulation. **(B)** Heat map of array-wide response to visual stimuli (white bars) before (left) and after (right) application of suction. **(C)** Histogram of observed spike amplitudes (normalized to units of RMS noise) before (gray) and after (red) application of suction.

In contrast to the brain slices, retinae exhibited ample activity on many electrodes without suction, both spontaneously and in response to flashes of light (Figure 4A, left). Following suction, however, both the number of spikes and their SNR increased (Figure 4A, right). The increase in recorded firing rate was substantial on the great majority of electrodes (Figure 4B), and the fraction of spikes with SNR above 10 increased by 56% (Figure 4C).

### 3.4. Isolated leech ganglia

**Figure 5:**
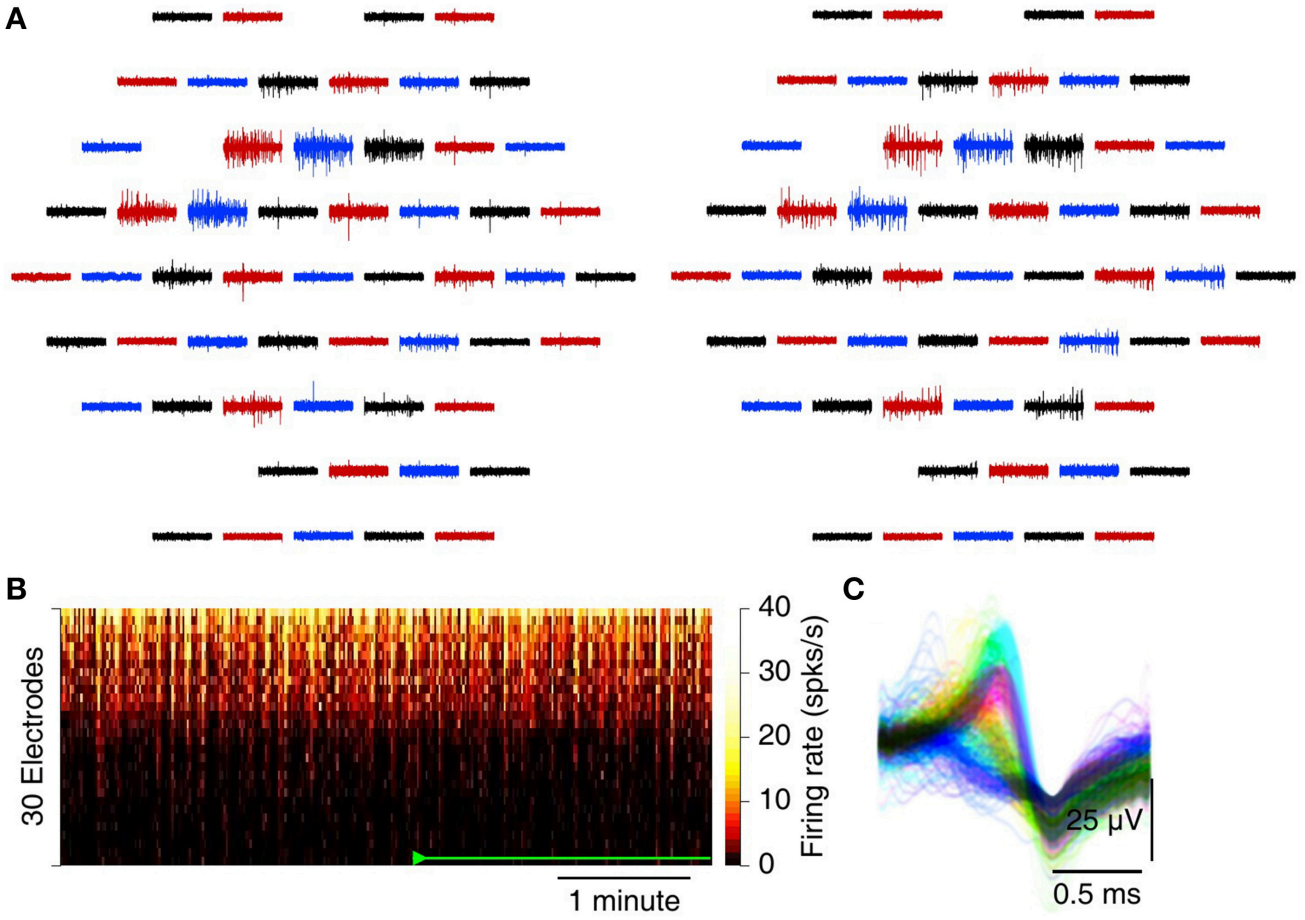
**Recording spontaneous activity from an isolated leech ganglion with an MSEA**. **(A)** Simultaneously recorded traces from 60 electrodes before (left) and after (right) application of 32 mbar of suction. Each trace represents data from one electrode; traces in the graph are laid out in the same geometry as electrodes in the array. Colors only serve to guide the eye. **(B)** Heat map of firing rates on each of 30 electrodes (sorted in order of total spike count) with and without suction. Suction 8.3 mbar was applied after the start of the recording (green marks). **(C)** Example from a single electrode of the waveforms of all spikes. Colors: units as identified by UltraMegaSort2000 (see Section 2).

The leech segmental ganglion has been a popular preparation for neuroscientists because of the direct mapping between the activity of readily identifiable neurons and specific behaviors (Kristan et al., 2005). Furthermore, the accessibility of the leech nervous system makes it an excellent test-bed for new technologies (Ferguson et al., 2012; Miller et al., 2012). Nevertheless, we are not aware of any previous publications using MEAs to record from these ganglia. We found that MSEA recordings from an isolated leech ganglion displayed ample spontaneous activity, but that the activity did not appreciably change after applying suction (Figure 5).

### 3.5. Analysis of detected spikes

**Figure 6:**
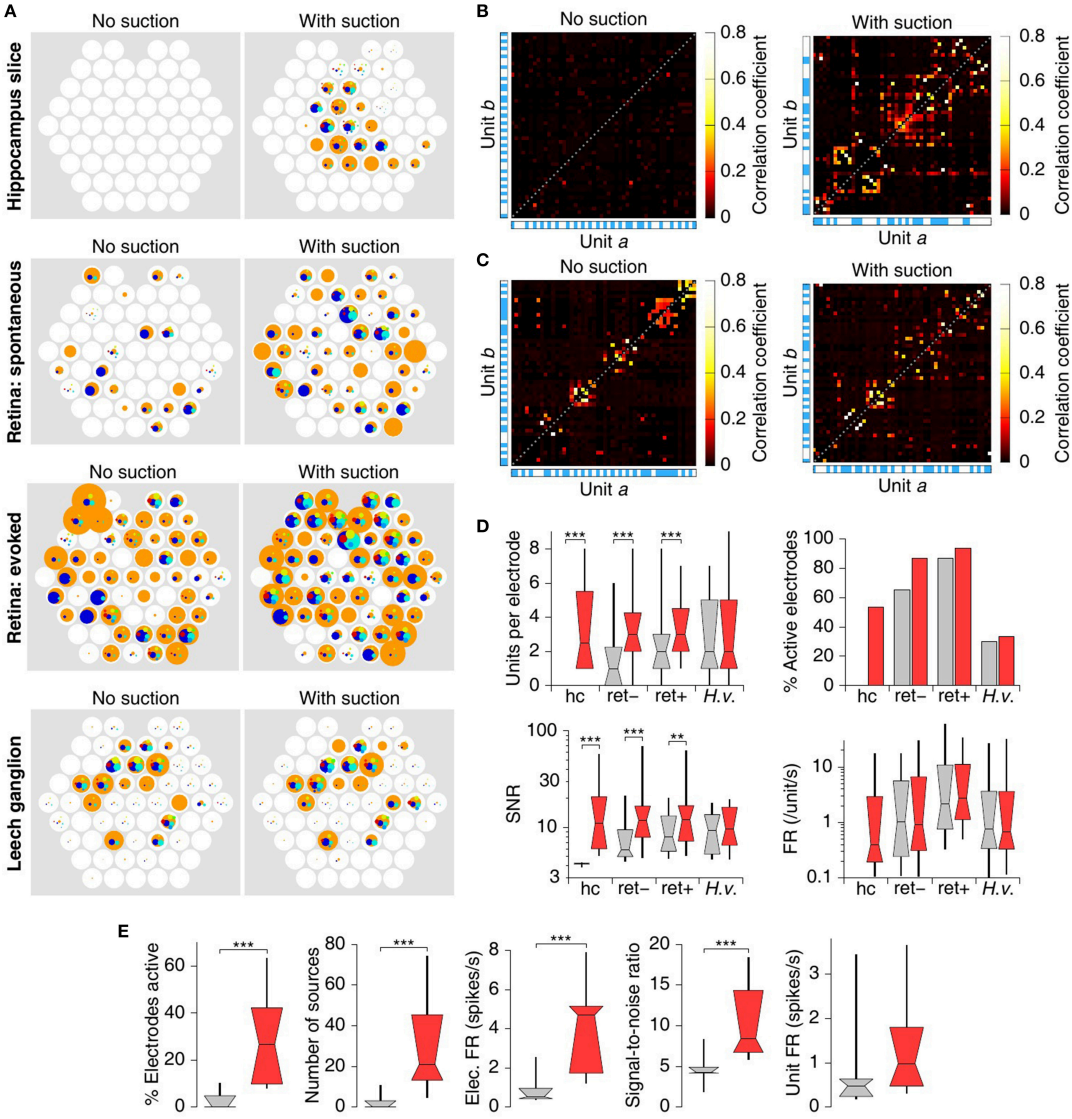
**Analysis of detected spikes**. **(A)** Number of sources per electrode and firing rate for each source in a cortico-hippocampal slice, a retina, and a leech ganglion (top to bottom), before and after application of suction (left and right). Each white circle represents an electrode, colored circles represent sources identified on that electrode. The area of the circles represent the firing rate of the corresponding source; a circle with the same area as the underlying white circles represents a firing rate of 20 spikes per second. **(B)** Cross correlations between the firing rates of the various sources in the slice of **(A)**. The alternating white-and blue stripes along the sides of the graphs indicate different electrodes. Only the 50 sources with strongest mutual cross correlations are shown in the graph. **(C)** Cross correlations between the firing rates of the various sources in the spontaneous retina recording of **(A)**. **(D)** Summary statistics of the number of isolated sources per electrode, the percentage electrodes that was active (see text), the signal-to-noise ratio of the strongest unit on each electrode, and the firing rates of all isolated units; in cortico-hippocampal slice (“hc”), retina in the dark (“ret–”), stimulated retina (“ret+”), and leech ganglion (“H.v.”). Boxes and whiskers indicate the 10th, 25th, 50th, 75th, and 90th percentiles of the distributions. Stars indicate significance: ^**^p < 0.01, ^***^p < 0.001; Mann–Whitney U-test. Note that activity in the hippocampus recording was minimal prior to suction, resulting in several invisible bars in this plot. **(E)** Comparison of spike detection with (red) and without (gray) suction in *N* = 15 cortico-hippocampal slices. From left to right: Percentage of electrodes that had at least one source with a firing rate above 0.1 Hz and SNR above 5; The total number of such sources across all electrodes; the mean firing rate across all usable electrodes; the mean across all usable electrodes of the signal-to-noise ratio of the strongest unit on that electrode; the mean across units of the rate of spikes attributable to that unit. ^***^p < 0.001; sign tests. Whiskers extend to 10th and 90th percentiles; bars show 25th, 50th, and 75th percentiles.

Spike-sorted results for all specimens are summarized in Figure 6. The number of isolated units (putative neurons) per electrode and firing rate for each unit in a cortico-hippocampal slice, a retina, and a leech ganglion before and after application of suction are represented spatially at the location of their respective recording electrodes in Figure 6A. Applying suction reveals substantial long-distance temporal correlations in the activity of neurons within the slice (Figure 6B). In the retina explant, such correlations are evident even before suction, but suction does increase their salience (Figure 6C). In the leech ganglion, it was common for a single source to produce spikes on multiple electrodes (data not shown). This made it challenging to perform a meaningful correlation analysis.

In all but the ganglion preparation, the average number of units isolated per electrode and the SNR of the “strongest” unit on each electrode increased significantly following suction. The number of active electrodes (those that had at least one isolated unit with SNR at least 5 × RMS noise) also increased in all cases (Figure 6D). By contrast, the firing rate of isolated units did not change, which is expected if suction does not alter the activity patterns of individual neurons and if spike sorting is correct. Note, though, that the unit firing rate before and after suction is not quite an “apples-to-apples” comparison, as many new sources are revealed by suction.

Overall, in 15 recordings from cortico-hippocampal slices, 3 ± 5% (mean ± SD) of electrodes were active before suction vs. 30 ± 20% after. Since the data were strongly non-normally distributed (Shapiro–Wilk test, *p* < 0.0001 and *p* < 0.02, respectively), we tested non-parametrically for differences that followed the application of suction; these were highly significant (Figure 6E; *p* < 0.001, sign test). This increase in number of active electrodes was accompanied by commensurate significant increases in the total number of sources isolated in the arrays, in the total number of spikes detected per unit time, and the signal-to-noise ratio of the spikes from the strongest unit for each electrode. The firing rates for isolated units did not increase significantly. (If the trend of a slight increase is real, it may stem from spikes from units with relatively low SNR gaining detectability after suction improved that SNR.) No significant differences were seen in the performance of circumferential vs. intercalated arrays; these were therefore analyzed together.

### 3.6. Cause of the improvements in recording quality

**Figure 7:**
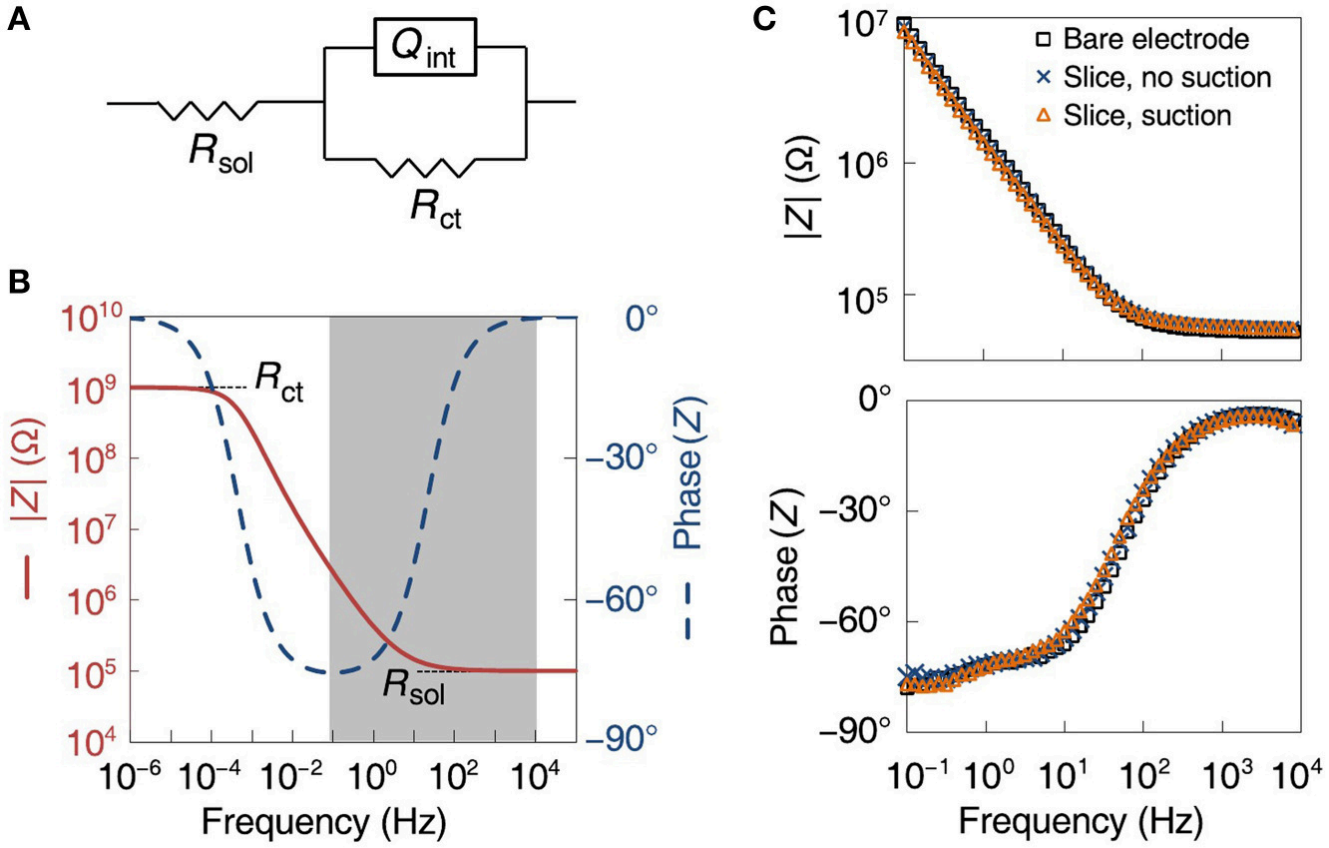
**Analysis of electrode-tissue interface**. **(A)** Electrical equivalence model of electrode and solution with circuit elements: *R*_*sol*_ (solution resistance), *Q*_*int*_ (constant phase angle impedance representing the interface capacitance), *R*_*ct*_ (electrode charge transfer resistance). **(B)** Theoretical model of absolute value (red) and phase (blue, dashed) of electrode impedance as a function of frequency. **(C)** Actual measurements of absolute value (top) and phase (bottom) of electrode impedance, before a slice was placed on the array (black squares), with a slice present but before suction was applied (blue crosses), and with a slice with suction applied (red triangles).

In order to determine the mechanism of increased recorded activity, we modeled the electrode–saline interface as a solution resistance in series with a parallel combination of the electrode charge transfer resistance and a constant phase angle impedance representing the interface capacitance, with typical values for the circuit components (see Section 2; Franks et al., 2005; Figure 7A). In the model, the solution resistance dominates the impedance at frequencies above 100 Hz, whereas the charge transfer resistance dominates at frequencies below 10^−4^ Hz. The interface capacitance controls the frequency ranges of these different regimes (Figure 7B). We then measured electrochemical impedance spectra from the MSEA electrodes before and after suction, over a frequency range of 0.1 Hz to 10 kHz. Compared to the situation when the MSEA was merely covered with saline, laying down a slice on it increased the electrode impedance by 3.1 ± 4.2% (at 1 kHz, *N* = 6 electrodes), whereas applying suction reduced it by 0.5 ± 14%. Changes in the phase of the impedance were similarly small, indicating no significant change in interface capacitance (Figure 7C). As these changes fell within one standard deviation of repeated impedance measurements of a single bare electrode (27%), it appears that suction did not enhance the electrode–cell membrane coupling *per se*. We hypothesize that the observed improvements resulted from the sources being pulled closer to the electrodes.

### 3.7. Compatibility with optical methods

**Figure 8:**
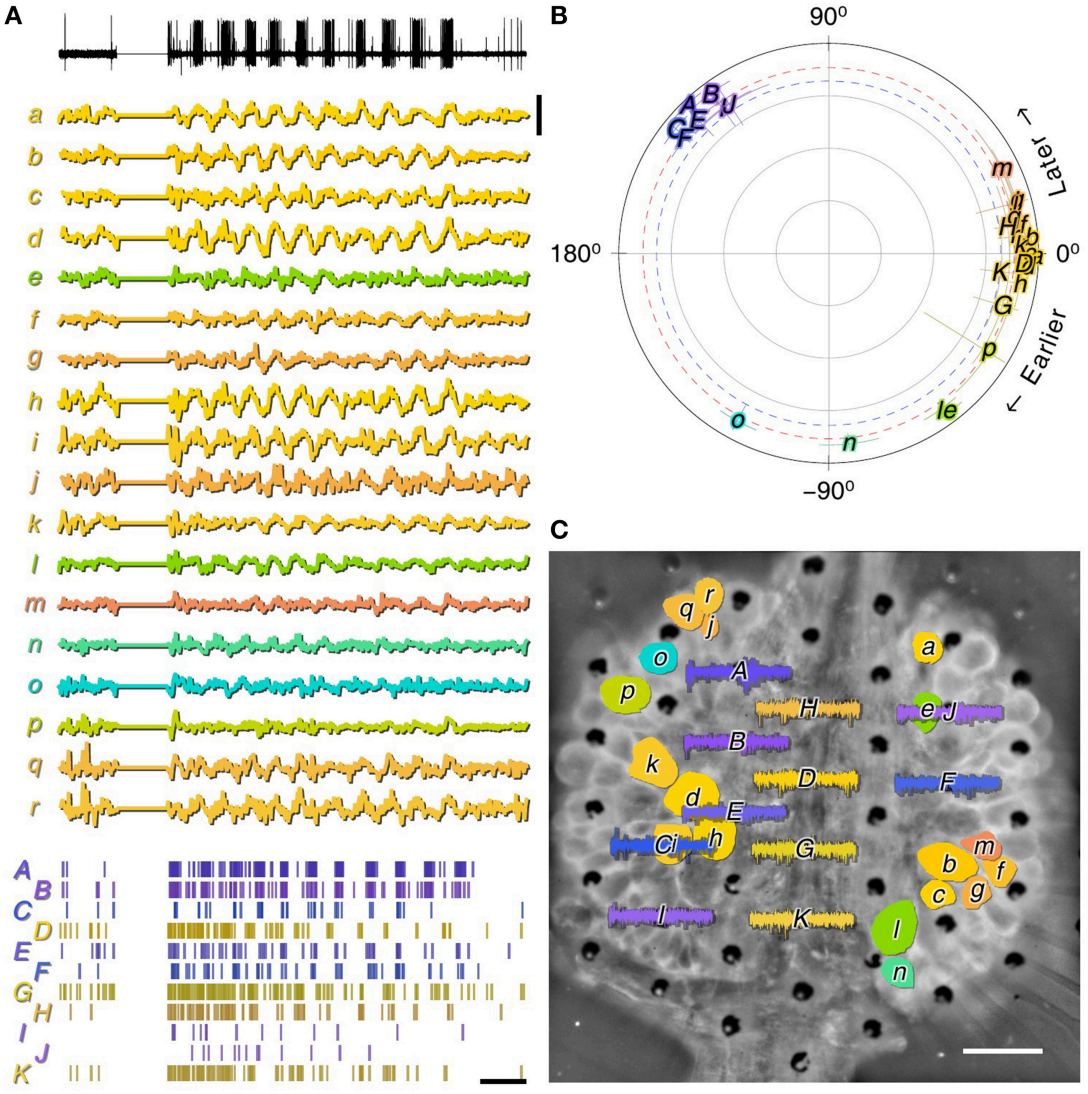
**MSEA and VSD recordings from *ex-vivo* leech ganglion**. **(A)** Activity recorded during fictive swimming simultaneously from a nerve that innervates dorsal longitudinal muscles using a suction electrode (black, top); from somata in the ganglion using a voltage sensitive dye (lower case labels, middle); and extracellularly in the ganglion using the MSEA (capitalized labels, bottom). Displayed signals are electrode voltage data (top); normalized VSD fluorescence (middle); and detected spikes (bottom). Scale bars: 0.2% dF/F, 2 s. **(B)** Coherence of VSD (lower case letters) and MSEA signals with respect to the nerve signal. Red and blue dotted lines are thresholds of significance for VSD and MSEA signals, respectively. **(C)** VSD and MSEA signals overlaid on a fluorescent image of the ganglion obtained by imaging through the MSEA. Scale bar: 100 μm. Colors indicate phase lag of signals relative to swim rhythm in all panels.

A critical motivation for developing MSEAs on a transparent quartz substrate was to enable the combination of multisite extracellular recording with optical methods. To demonstrate compatibility with optical methods, we isolated most of the central nervous system from a medicinal leech and applied the voltage-sensitive dye VF2.1.Cl to one ganglion in the middle of the nerve cord (see Section 2.7). This ganglion was then placed on the MSEA and we imaged VSD signals through the MSEA. Fictive swimming was evoked by electrically stimulating the dorsal posterior nerve root of the imaged ganglion through a glass suction electrode. Characteristic swim oscillations (Kristan et al., 2005) could readily be discerned in the recording from this electrode (Figure 8A, black trace). The VSD record contained many traces of identifiable neurons that participated in the swim rhythm at various phase delays (Figure 8A, colored traces). Simultaneously recorded spiking activity from many MSEA electrodes likewise was modulated along with the swim rhythm (Figure 8A, rasters). The spike trains clearly comprised two groups, one from putative neurons that were active in phase with the root nerve recording, and one at a 120° phase lag (Figure 8B, capitals). The neurons in the VSD record exhibited slightly more diverse phase lags (Figure 8B, lower case letters), indicating that the neuronal population sampled by the MSEA overlaps with but is not identical to the neurons that are visible in the VSD image (Figure 8C).

## 4. Discussion

Multi-suction electrode arrays (and perforated MEAs) have the potential to improve the electrical contact between neuronal sources and electrodes and to improve tissue health by increased perfusion with oxygenated medium.

We saw the most dramatic improvement in recordings from mouse brain slices. These produced almost no recordable spiking activity prior to suction, yet revealed copious activity after suction. In experiments with standard MEAs, researchers commonly use miniature “harps” comprising a metal ring with several nylon strings to press the slice down onto the MEA so as to ensure tight contact between slice and electrodes. While we could not dispense with harps entirely, to keep the slice from floating away prior to suction, we were able to rearrange the geometry so that the harp “strings” remained far from the recording area (Figure S1) which reduces the potential of tissue damage where it matters most and improves access to the tissue for simultaneous intracellular recording.

The improvement in recording quality suggests that either the tissue–electrode interface has changed, or neuronal sources are brought closer to the electrodes, or both. Previous studies found that culturing rat neurons on semiconductor electrodes for 2–3 days resulted in an increase of the sheet resistance between the neuronal membrane and electrode surface to 14 MΩ on average. On the basis of imaging experiments, this was ascribed to a cleft forming between the membrane and electrode. The cleft was approximately 50 nm in height and the liquid in it has a resistivity close to that of bulk saline (Braun and Fromherz, 2004). Similar results have also been observed with cells cultured on gold electrodes (Lo and Ferrier, 1998). However, on the basis of electrochemical impedance spectra, we ruled out a change in interface impedance in our experiments (Figure 7C), indicating the slice is effectively electrically “transparent.” Two complementary mechanisms may provide an explanation for the discrepancy with earlier studies. First, the transistor and “bright” gold electrodes used in the cited studies are essentially flat in contrast to the platinum black electrodes used here, which are composed of nanoscale “ruffles” (Figure 2, step 8). Thus, even if a slice touches our electrodes, the majority of the electrode surface area most likely is not directly covered by a cell membrane. Second, brain slices contain many dead neurons and cellular debris on their surfaces (Buskila et al., 2014), which may not favor large single neurons annealing onto the electrode surface. In any event, such annealing would not be expected to occur in the short time (30 min) between the moment we laid the brain slice onto the electrode array and the start of recordings. These observations imply that the improvement in recording quality following applied suction through the MSEA is a result of the neuronal sources being pulled closer to the electrodes.

Compared to the brain slices, the retina explant showed less dramatic but still highly significant improvements in recording quality following application of suction (Figure 6D). The fact that the retina produced more recordable activity before suction than the brain slice may be due to the fact that the retina preparation does not have a layer of dead cells on its surface, or because we started out with greater pressure from above: The retina explant is curved, as it fits the eye cup *in vivo*. Typically, retinae are pushed onto a MEA with a screw-like contraption. Alternatively, with perforated MEAs, filter paper can be used to keep the retina flat before applying suction (Reinhard et al., 2014). We used a custom-built harp with wide-nylon strings that applied minimal pressure to keep the retina almost flat and just barely touching the array prior to suction. While we obtained ample activity from the retina before suction, we still saw a 72% increase of detectable spikes following suction, and a nearly three-fold increase in spikes with SNR over 10. It has been hypothesized that pulling the tissue toward the array using suction rather than pushing it toward the array using applied pressure may be beneficial to tissue integrity (Reinhard et al., 2014). Indeed, we could record strong signals from this preparation for at least 4.5 h.

In the slice and especially the retina experiments, activation of mechanosensory channels could be a contributing factor to the increase in recorded activity (Tan et al., 2006). In particular, such a mechanism could explain part of the transient increase in activity at the moment suction was applied. However, it is not likely that mechanosensory channels are a major factor in the sustained effect of suction: In both brain slice and retina, the number of recorded units and their SNR increased greatly following suction, whereas the firing rates of individual cells increased little if at all (Figures 6D,E).

The leech ganglia recordings benefited least from suction. When recording from leech ganglia, we obtained fewer active electrodes and fewer isolated spiking units than from the other preparations, and applying suction had minimal effect (Figure 5). This may have to do with the structure of the ganglion, which comprises a layer of cell bodies surrounding a central neuropil. After the enclosing sheath is removed—which is required to record any activity—the cell bodies are not very tightly held in an extracellular matrix. Accordingly, it was necessary to physically press the ganglion down onto the array, because suction—or even continuous flow through the suction pores—was insufficient to hold the tissue down. Our MSEA recordings contained around 10 sources that oscillated rhythmically with swimming. This is a small fraction of the total population of neurons known to oscillate in this manner (Briggman and Kristan, 2006). One possible explanation is that leech neurons generate action potentials at some distance from their cell bodies at specific spike initiation zones located along the neurite. From there, action potentials only passively propagate into the soma. In our setup the somata were closest to the electrode array, and it is possible that somatic depolarizations were simply too small or too slow to result in a detectable signal on the electrodes. Additionally, debris from the membranes of large glial cells covers the ganglion even after desheathing (Kuffler and Potter, 1964) which may further distort signal propagation between individual neurons and the recording electrodes (Figure S3).

Recording electrical activity from many neurons at once with single-spike resolution is an extremely powerful capability. While progress is being made to do this with VSDs alone (Moshtagh-Khorasani et al., 2013), sensitivity and the speed of VSD responses remain limiting factors in many experimental situations. Additionally, phototoxicity limits the total amount of recording time available before tissue health degrades to an unacceptable degree. Combining VSD imaging with MSEA recording has the potential of overcoming this limitation: By correlating spike trains obtained from the MSEA with activity obtained from the VSD in a modest amount of recording time, the cellular identity of the sources of MSEA activity can be established. After that, the MSEA can be used to obtain recordings of practically unlimited duration of those cells' spiking activity. Our current experiments on the leech nervous system (Figure 8) come part of the way toward attaining this potential. Importantly, they demonstrate that MSEAs are fully compatible with VSD imaging. There is no reason why this combination could not be used to great advantage on brain slices. In that same context, the transparent MSEAs' implied compatibility with optogenetic stimulation (Häusser, 2014), combined with its enhanced electrical recording capability, opens up many future research opportunities.

## Author contributions

JN and DW conceived and designed the project. JN and RP performed cortico-hippocampal slice experiments. JN, DW, and HA performed retina experiments. JN, AS, and DW performed leech experiments. DW performed the spike sorting and coherence analysis. JN and DW analyzed data and wrote the manuscript.

## Funding

Funding for this work was provided by the Broad Foundations, by a Career Award at the Scientific Interface (#1007977) from the Burroughs Wellcome Fund to DW, and by funds provided by the Regents of the University of California, Tobacco-Related Diseases Research Program (grant no 22DT-0008) to RP.

### Conflict of interest statement

The authors declare that the research was conducted in the absence of any commercial or financial relationships that could be construed as a potential conflict of interest.

## References

[B1] Baljon P. L. Wagenaar D. A. (2015). Responses to conflicting stimuli in a simple stimulus-response pathway. J. Neurosci. 35, 2398–2406. 10.1523/JNEUROSCI.3823-14.2015 25673834 PMC4323524

[B2] Braun D. Fromherz P. (2004). Imaging neuronal seal resistance on silicon chip using fluorescent voltage-sensitive dye. Biophys. J. 87, 1351–1359. 10.1529/biophysj.104.039990 15298937 PMC1304473

[B3] Briggman K. L. Kristan W. B. Jr. (2006). Imaging dedicated and multifunctional neural circuits generating distinct behaviors. J. Neurosci. 26, 10925–10933. 10.1523/JNEUROSCI.3265-06.2006 17050731 PMC6674766

[B4] Buskila Y. Breen P. P. Tapson J. van Schaik A. Barton M. Morley J. W. (2014). Extending the viability of acute brain slices. Sci. Rep. 4:5309. 10.1038/srep05309 24930889 PMC4058870

[B5] Cacciatore T. W. Brodfuehrer P. D. Gonzalez J. E. Jiang T. Adams S. R. Tsien R. Y.. (1999). Identification of neural circuits by imaging coherent electrical activity with FRET-based dyes. Neuron 23, 449–459. 10433258 10.1016/s0896-6273(00)80799-0

[B6] Eaton J. W. Bateman D. Hauberg S. Wehbring R. (2014). The GNU Octave 3.8 Reference Manual: Free Your Numbers. Samurai Media Limited. Available online at: http://www.gnu.org/software/octave/doc/interpreter

[B7] Egert U. Okujeni S. Nisch W. Boven K. H. Rudorf R. Gottschlich N. (2005). Perforated microelectrode arrays optimize oxygen availability and signal-to-noise ratio in brain slice recordings, in Mikrosystemtechnologie Kongress, eds Zengerle R. Fabricius N. Gessner T. (Freiburg: VDE Verlag), 431–434.

[B8] Fee M. S. Mitra P. P. Kleinfeld D. (1996). Automatic sorting of multiple unit neuronal signals in the presence of anisotropic and non-Gaussian variability. J. Neurosci. Methods 69, 175–188. 8946321 10.1016/S0165-0270(96)00050-7

[B9] Ferguson J. E. Boldt C. Puhl J. G. Stigen T. W. Jackson J. C. Crisp K. M.. (2012). Nanowires precisely grown on the ends of microwire electrodes permit the recording of intracellular action potentials within deeper neural structures. Nanomedicine 7, 847–853. 10.2217/nnm.11.157 22475650 PMC3774295

[B10] Franks W. Schenker I. Schmutz P. Hierlemann A. (2005). Impedance characterization and modeling of electrodes for biomedical applications. IEEE Trans. Biomed. Eng. 52, 1295–1302. 10.1109/TBME.2005.847523 16041993

[B11] Gaudry Q. Kristan W. B. Jr. (2012). Decision points: the factors influencing the decision to feed in the medicinal leech. Front. Neurosci. 6:101. 10.3389/fnins.2012.00101 22783162 PMC3390556

[B12] Gross G. W. (1979). Simultaneous single unit recording *in vitro* with a photoetched laser deinsulated gold multimicroelectrode surface. IEEE Trans. Biomed. Eng. 26, 273–279. 447356 10.1109/tbme.1979.326402

[B13] Gross G. W. Rieske E. Kreutzberg G. W. Meyer A. (1977). A new fixed-array multi-microelectrode system designed for long-term monitoring of extracellular single unit neuronal activity *in vitro*. Neurosci. Lett. 6, 101–105. 19605037 10.1016/0304-3940(77)90003-9

[B14] Gross G. W. Wen W. Y. Lin J. W. (1985). Transparent indium-tin oxide electrode patterns for extracellular, multisite recording in neuronal cultures. J. Neurosci. Methods 15, 243–252. 4094480 10.1016/0165-0270(85)90105-0

[B15] Harley C. M. Cienfuegos J. Wagenaar D. A. (2011). Developmentally regulated multisensory integration for prey localization in the medicinal leech. J. Exp. Biol. 214, 3801–3807. 10.1242/jeb.059618 22031745

[B16] Häusser M. (2014). Optogenetics: the age of light. Nat. Methods 11, 1012–1014. 10.1038/nmeth.3111 25264778

[B17] Hill D. N. Mehta S. B. Kleinfeld D. (2011). Quality metrics to accompany spike sorting of extracellular signals. J. Neurosci. 31, 8699–8705. 10.1523/JNEUROSCI.0971-11.2011 21677152 PMC3123734

[B18] Inoué T. Osatake H. (1988). A new drying method of biological specimens for scanning electron microscopy: the t-butyl alcohol freeze-drying method. Arch. Histol. Cytol. 51, 53–59. 3137948 10.1679/aohc.51.53

[B19] Kristan W. B. Stent G. S. Ort C. A. (1974). Neuronal control of swimming in medicinal leech. 1. Dynamics of swimming rhythm. J. Comp. Physiol. 94, 97–119.

[B20] Kristan W. B. Jr. Calabrese R. L. Friesen W. O. (2005). Neuronal control of leech behavior. Prog. Neurobiol. 76, 279–327. 10.1016/j.pneurobio.2005.09.004 16260077

[B21] Kuffler S. W. Potter D. D. (1964). Glia in the leech central nervous system: physiological properties and neuron-glia relationship. J. Neurophysiol. 27, 290–320. 14129773 10.1152/jn.1964.27.2.290

[B22] Lefebvre J. L. Zhang Y. Meister M. Wang X. Sanes J. R. (2008). gamma-Protocadherins regulate neuronal survival but are dispensable for circuit formation in retina. Development 135, 4141–4151. 10.1242/dev.027912 19029044 PMC2644426

[B23] Lo C. M. Ferrier J. (1998). Impedance analysis of fibroblastic cell layers measured by electric cell-substrate impedance sensing. Phys. Rev. E 57, 6982.

[B24] Maher M. P. Pine J. Wright J. Tai Y. C. (1999). The neurochip: a new multi-electrode device for stimulating and recording from cultured neurons. J. Neurosci. Methods 87, 45–56. 10065993 10.1016/s0165-0270(98)00156-3

[B25] Meister M. Wong R. O. L. Baylor D. A. Shatz C. J. (1991). Synchronous bursts of action potentials in ganglion cells of the developing mammalian retina. Science 252, 939–943. 2035024 10.1126/science.2035024

[B26] Miller E. W. Lin J. Y. Frady E. P. Steinbach P. A. Kristan W. B. Jr. Tsien R. Y. (2012). Optically monitoring voltage in neurons by photo-induced electron transfer through molecular wires. Proc. Natl. Acad. Sci. U.S.A. 109, 2114–2119. 10.1073/pnas.1120694109 22308458 PMC3277584

[B27] Moshtagh-Khorasani M. Miller E. W. Torre V. (2013). The spontaneous electrical activity of neurons in leech ganglia. Physiol. Rep. 1:e00089. 10.1002/phy2.89 24303164 PMC3841027

[B28] Nagarah J. M. Paek E. Luo Y. Wang P. Hwang G. S. Heath J. R. (2010). Batch fabrication of high-performance planar patch-clamp devices in quartz. Adv. Mater. 22, 4622–4627. 10.1002/adma.201001793 20830714

[B29] Nagarah J. M. Wagenaar D. A. (2012). Ultradeep fused silica glass etching with an HF-resistant photosensitive resist for optical imaging applications. J. Micromech. Microeng. 22:035011 10.1088/0960-1317/22/3/035011

[B30] Obien M. E. Deligkaris K. Bullmann T. Bakkum D. J. Frey U. (2014). Revealing neuronal function through microelectrode array recordings. Front. Neurosci. 8:423. 10.3389/fnins.2014.00423 25610364 PMC4285113

[B31] Peterka D. S. Takahashi H. Yuste R. (2011). Imaging voltage in neurons. Neuron 69, 9–21. 10.1016/j.neuron.2010.12.010 21220095 PMC3387979

[B32] Pine J. (1980). Recording action potentials from cultured neurons with extracellular microcircuit electrodes. J. Neurosci. Methods 2, 19–31. 7329089 10.1016/0165-0270(80)90042-4

[B33] Reinhard K. Tikidji-Hamburyan A. Seitter H. Idrees S. Mutter M. Benkner B.. (2014). Step-by-step instructions for retina recordings with perforated multi electrode arrays. PLoS ONE 9:e106148. 10.1371/journal.pone.0106148 25165854 PMC4148441

[B34] Ryan T. J. Kopanitsa M. V. Indersmitten T. Nithianantharajah J. Afinowi N. O. Pettit C.. (2013). Evolution of GluN2A/B cytoplasmic domains diversified vertebrate synaptic plasticity and behavior. Nat. Neurosci. 16, 25–32. 10.1038/nn.3277 23201971 PMC3979286

[B35] Steidl E. M. Neveu E. Bertrand D. Buisson B. (2006). The adult rat hippocampal slice revisited with multi-electrode arrays. Brain Res. 1096, 70–84. 10.1016/j.brainres.2006.04.034 16716268

[B36] Tan J. C. Kalapesi F. B. Coroneo M. T. (2006). Mechanosensitivity and the eye: cells coping with the pressure. Br. J. Ophthalmol. 90, 383–388. 10.1136/bjo.2005.079905 16488967 PMC1856976

[B37] Taylor A. L. Cottrell G. W. Kleinfeld D. Kristan W. B. Jr. (2003). Imaging reveals synaptic targets of a swim-terminating neuron in the leech CNS. J. Neurosci. 23, 11402–11410. 14673004 10.1523/JNEUROSCI.23-36-11402.2003 PMC6740517

[B38] Tye K. M. Deisseroth K. (2012). Optogenetic investigation of neural circuits underlying brain disease in animal models. Nat. Rev. Neurosci. 13, 251–266. 10.1038/nrn3171 22430017 PMC6682316

[B39] Wagenaar D. A. (2012). An optically stabilized fast-switching light emitting diode as a light source for functional neuroimaging. PLoS ONE 7:e29822. 10.1371/journal.pone.0029822 22238663 PMC3253093

[B40] Wagenaar D. A. (2014). Publication quality 2D graphs with less manual effort due to explicit use of dual coordinate systems. Source Code Biol. Med. 9:22 10.1186/1751-0473-9-22

[B41] Wagenaar D. A. DeMarse T. B. Potter S. M. (2005). MEABench: a toolset for multi-electrode data acquisition and on-line analysis, in Proceedings of 2nd International IEEE EMBS Conference on Neural Engineering, eds Akay M. Wolf L. J. Stock J. (Washington, DC), 518–521.

[B42] Wagenaar D. A. Pine J. Potter S. M. (2006). An extremely rich repertoire of bursting patterns during the development of cortical cultures. BMC Neurosci. 7:11. 10.1186/1471-2202-7-11 16464257 PMC1420316

