# Supplementary material for: Optically transparent multi-suction electrode arrays
Source: Front Neurosci. 2015 Oct 20;9:384. doi: 10.3389/fnins.2015.00384 (PMC4611137; doi:10.3389/fnins.2015.00384)
Supplement: Supplementary file 1 [file Presentation1.PDF]

Optically transparent multi–suction electrode arrays

John M. Nagarah, Annette Stowasser, Rell L. Parker, Hiroki Asari, and Daniel A. Wagenaar

Supplementary figures

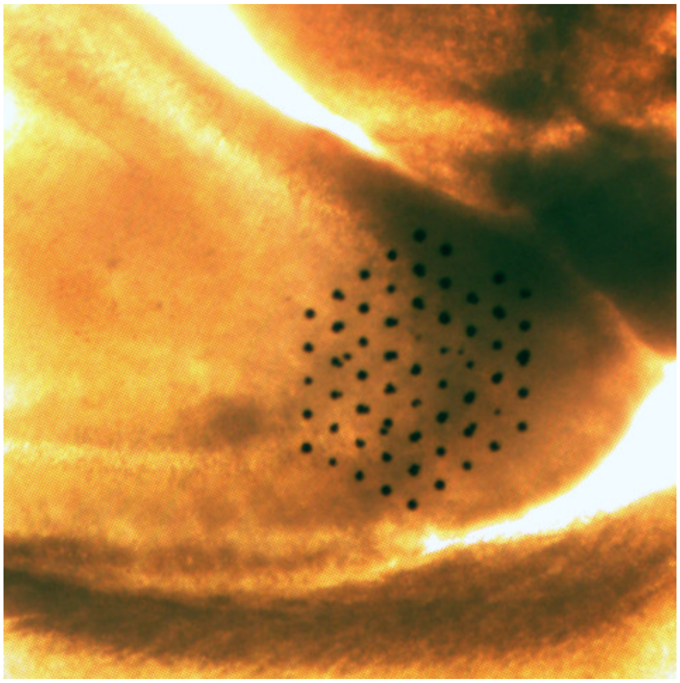

Figure S1. Photograph of a cortico-hippocampal slice on a MSEA. The CA3 region of the hippocampus is positioned over the electrode array.

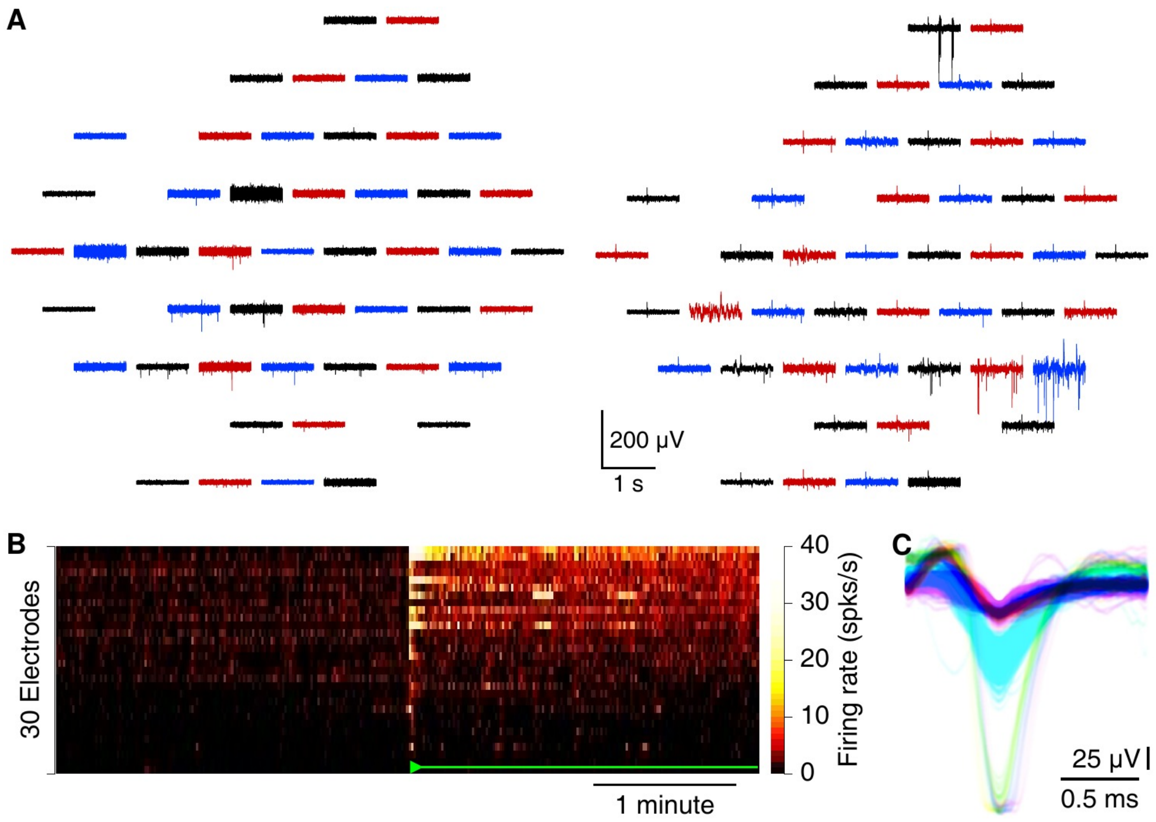

Figure S2. Recording spontaneous activity from cortico-hippocampal slices with an MSEA with a circumferential electrode design. **A.** Simultaneously recorded traces from 60 electrodes before (left) and after (right) application of 120 mbar of suction. Each trace represents data from one electrode; traces in the graph are laid out in the same geometry as electrodes in the array. Colors only serve to guide the eye. **B.** Heat map of firing rates on each of 30 electrodes (sorted in order of total spike count) with and without suction. Suction was applied 5 minutes after the start of the recording (green marks). **C.** Example from a single electrode of the waveforms of all spikes. Colors: units as identified by UltraMegaSort2000 (see Online Methods).

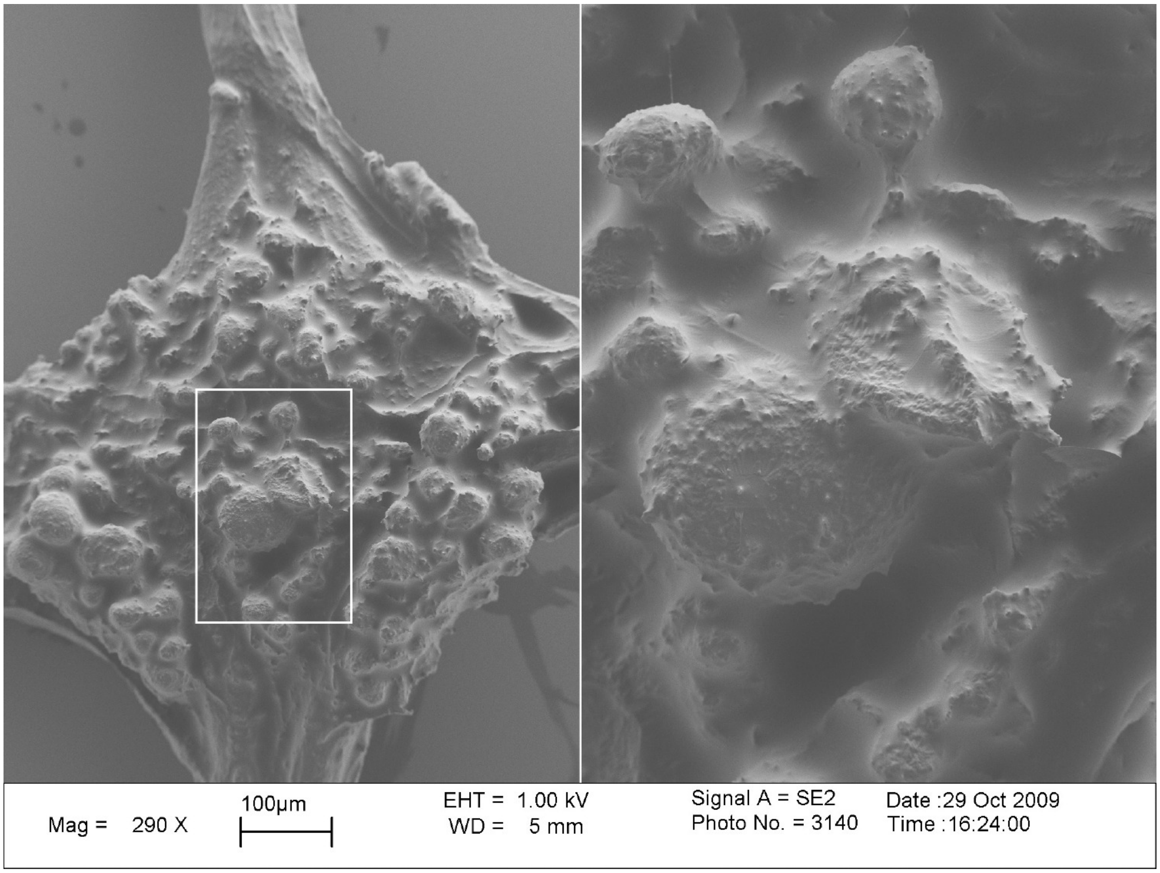

Figure S3. SEM image of a fixed leech ganglion. Glial membrane can be seen draping over neurons in the leech ganglion after desheathing.
